# Comparison of the efficacy of intravitreal ranibizumab for choroidal neovascularization due to pathological myopia with and without a dome-shaped macula

**DOI:** 10.1097/MD.0000000000009251

**Published:** 2017-12-15

**Authors:** Bincui Cai, Jin Yang, Shuang Li, Linni Wang, Lu Chen, Xiaorong Li, Zhiqing Li

**Affiliations:** Tianjin Medical University Eye Hospital, Tianjin, China.

**Keywords:** CNV, dome-shaped macula, OCT, pathologic myopia, ranibizumab

## Abstract

Ranibizumab injection in the treatment of choroidal neovascularization (CNV) secondary to pathologic myopia (PM) with and without a dome-shaped macula (DSM).

Prospective observational study.

A total of 24 patients (24 eyes) with angiographic evidence of CNV secondary to PM were divided into 2 groups: eyes with a DSM and eyes without DSM. All patients received a baseline intravitreal ranibizumab injection. Additional injections were considered at each follow-up visit. Best-corrected visual acuity (BCVA) and optical coherence tomography were tested monthly through 12 months of follow-up. The mean changes in BCVA, central retinal thickness (CRT, including retinal and CNV thickness), and the number of injections were evaluated.

There were no significant differences in visual outcomes between the groups over 12 months (*P* > .05). Patients with a DSM had a mean change in BCVA of +8.7 letters compared with +14.2 letters in patients without a DSM (*P* = .68). However, there were more patients without a DSM who gained at least 15 letters from baseline compared with patients with a DSM. By the end of the follow-up, there was no significant difference in the mean change in baseline CRT between patients with and without a DSM (−65.0 and −90.7, respectively, *P* = .42). The mean number of injections was 8.83 in the patients with DSM and 8.17 in the patients without a DSM (*P* > .05).

For the pathological myopia patients who had CNV with a DSM, the DSM did not alter the effect of the ranibizumab treatment. There was no difference in the visual improvement, anatomic benefit and number of treatments between the 2 groups.

## 1 Introduction

Pathologic myopia (PM) is one of the leading causes of vision loss in young people worldwide and is particularly prevalent in China.^[1]^ PM is defined as high myopia with various degenerative changes in the posterior segment structures associated with progressive and excessive elongation of the globe, which relates to several complications.^[2]^ These changes are related to complications such as macular hole (MH), maculoschisis choroidal neovascularization (CNV), and chorioretinal atrophy (CRA).^[3]^ Myopic choroidal neovascularization (mCNV) secondary to PM is one of the most common PM complications worldwide, especially in Asian populations. mCNV occurs in approximately 5.2% to 11.3% of pathological myopic eyes, causing central vision loss and affecting patients’ quality of life in their working years.^[4,5]^ Modalities for treating mCNV are argon laser for extrafoveal and juxtafoveal CNV and photodynamic therapy (PDT) for subfoveal CNV. For some patients, there is no effective treatment.^[6]^ The use of vascular endothelial growth factor inhibitors (anti-VEGF) is currently the first-line medical treatment for CNV. In addition, there is evidence that anti-VEGF therapy can improve vision acuity through delaying CRA.^[7]^

The dome-shaped macula (DSM) was first defined by Gaucher et al as an inward bulge of the macula in eyes with high myopia and staphyloma.^[8]^ In a recent study, Liang et al showed that the DSM was found in 225 of 1118 highly myopic eyes (20.1%), which suggests that a DSM is a frequent characteristic in highly myopic eyes. In addition, he concluded that the presence of a DSM was significantly associated with age and axial length through a multiple regression analysis. The patients with a DSM were younger and had longer axial length than the patients without a DSM.^[9]^ Two possible additional mechanisms for DSM formation were suggested: the first, vitreous traction contributes to the dome shape alteration; another explanation is hypotonia leads to the scleral wall collapsing inward, which may result in hypotony maculopathy.^[10]^

We consider that the changes of anatomical structure of eye posterior portion may affect the formation and change of CNV after ranibizumab treatment. Although CNV and DSM are both frequent complications of PM, the role of the DSM in the formation and treatment of CNV are still unknown. Previous studies reported CNV rates in patients with DSM, but there is no direct evidence showing that CNV is a unique complication to a DSM rather than a general feature of patients with PM.^[11,12]^ Therefore, we performed individual intravitreal ranibizumab therapy for patients having CNV secondary to PM, with and without a DSM, to investigate whether DSM alters the effect of the intravitreal ranibizumab therapy by comparing BCVA, CRT, and the number of injections.

## 2 Materials and methods

### 2.1 Patients

All the patients within our study were seen at Tianjin Medical University Eye Hospital from the beginning of September 2015 to the end of April 2016. We selected 24 highly myopic eyes of 24 patients diagnosed as having PM with active CNV. All selected patients gave informed consent to the study protocol, which was approved by the Ethics Committee of Tianjin Medical University Eye Hospital (Tianjin, China). The activity of CNV was assessed by fundus fluorescent angiography and indocyanine green angiography. Patients were eligible for inclusion in the study if they were at least 18 years of age and had active CNV secondary to PM. PM was characterized by a refractive error over −6.00 D and/or an axial length over 26.5 mm, accompanied by various complications in the fundus.^[5,7]^ The main exclusion criteria included the following: the presence of CNV with an origin other than PM, ocular inflammation, vitreoretinal surgery, and unclear optical coherence tomography (OCT) images.

**Figure 1:**
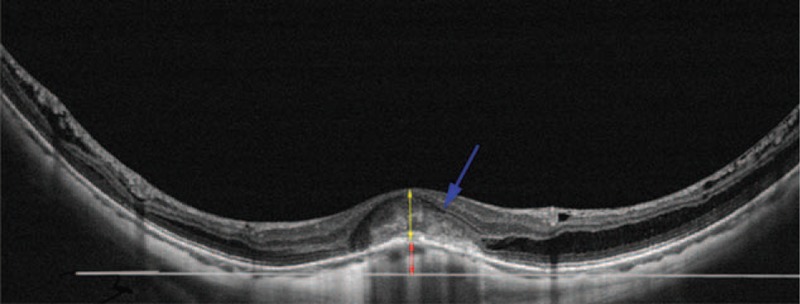
The dome-shaped macula detected by optical coherence tomography, showing a subretinal hyperreflectivity with mild intraretinal cysts (blue arrow). The central retinal thickness, including retinal and choroidal neovascularization thickness (yellow double arrow) was measured. The red double arrow showed the height of the inward bulge of the retinal pigment epithelium (RPE) above the tangent line of the RPE (white line) of the 2 outward concavities at the bottom of posterior staphyloma.

As used by Ellabban and Ohsugi, a DSM was defined as the presence of an inward bulge of the retinal pigment epithelium of more than 50 μm on the vertical or horizontal section of an OCT image^[11]^ (Fig. 1).

### 2.2 Examinations and measurements

All patients underwent a comprehensive baseline ocular examination including collection of demographic information, assessments of the best-corrected visual acuity (BCVA), refractive errors, intraocular pressure (IOP), axial length measurement using ocular biometry (IOLMaster; Carl Zeiss Meditec, Jena, Germany), and a spectral-domain OCT examination using Optovue OCT (Optovue, Fremont, CA). All eyes underwent a fluorescein angiography and indocyanine green angiography examination using Spectralis HRAt OCT (Heidelberg Engineering, Heidelberg, Germany). CNV size at baseline was measured by image J software.

At each follow-up visit, patients received complete ophthalmic assessments. All patients were evaluated for BCVA and central retinal thickness (CRT, including retinal and CNV thickness) (detected on the OCT) every month. BCVA was determined according to the Early Treatment Diabetic Retinopathy Study (ETDRS) charts. CRT was assessed by OCT. Additional treatments were considered if there was a BCVA loss of at least 5 ETDRS letters or any fluid on OCT, which is the gold standard for a treatment decision.

### 2.3 Statistical analysis

All statistical analyses were performed with SPSS for windows software (version 17.0, SPSS, Inc., Chicago, IL). In order to verify homogeneity, refractive error, IOP, axial length, BCVA and CRT in 2 groups were compared at baseline with the independent sample T test. Sex and ages in 2 groups were compared with the Fisher exact test. The mean change from baseline in BCVA and CRT in the 2 groups was analyzed using the independent sample T test. The mean number of injections in the 2 groups was analyzed using independent sample T test. *P* value < .05 was considered to indicate a significant difference.

## 3 Results

### 3.1 Descriptive data

The study included 24 eyes of 24 patients with CNV secondary to PM. Among the 24 eyes, 6 (25%) had a DSM that met our definition. The mean patient ages were 58.3 ± 5.0 years in the DSM (+) group and 57.8 ± 10.2 years in the DSM (−) group, with no significant differences. Among them, 7 were males and 17 were females. The mean refractive error of patients was −12.8 D (range, −22 to −6). The mean IOP was 14.2 mm Hg (range, 11–20). The baseline clinical characteristics of the 6 eyes with DSM and 18 eyes without DSM are summarized in Table 1.

**Table 1:**
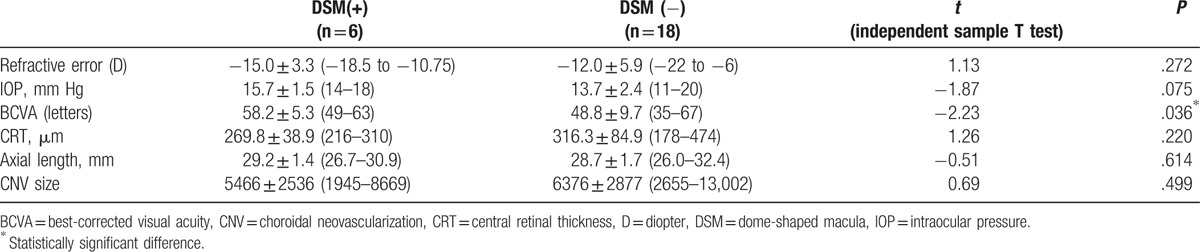
Baseline clinical characteristics of eyes with and without DSM.

### 3.2 Visual outcomes

**Figure 2:**
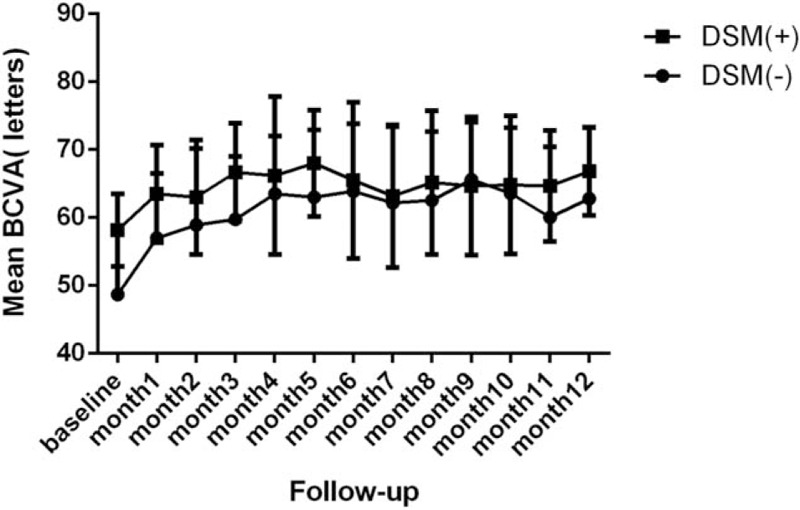
Mean best-corrected visual acuity (BCVA) in the DSM (+) and DSM (−) group during 12 months of follow-up. DSM = dome-shaped macula.

At baseline, the mean BCVA in the DSM (+) group and DSM (−) group was 58.2 ± 5.3 ETDRS letters and 48.8 ± 9.7 ETDRS letters, respectively. The BCVA in patients with and without DSM at month 12 was equally good (66.83 and 62.83 letters, respectively). BCVA changes during 12 months of follow-up are shown in Fig. 2.

**Figure 3:**
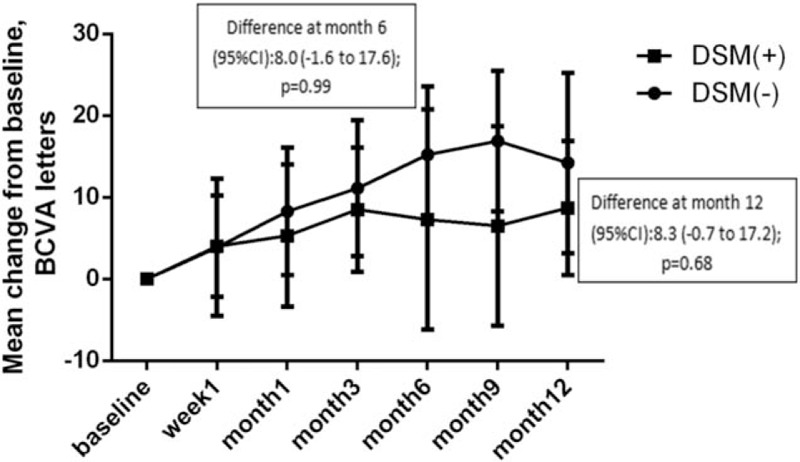
Mean change in best-corrected visual acuity (BCVA) from baseline to month 12: full analysis set. CI = confidence interval.

At month 6, the mean change in BCVA of patients with and without DSM was +7.3 letters and +15.2 letters, respectively (*P* = .99). At month 12, the mean change in BCVA of patients with and without DSM was +8.7 letters and +14.2 letters, respectively (*P* = .68) (Fig. 3).

**Figure 4:**
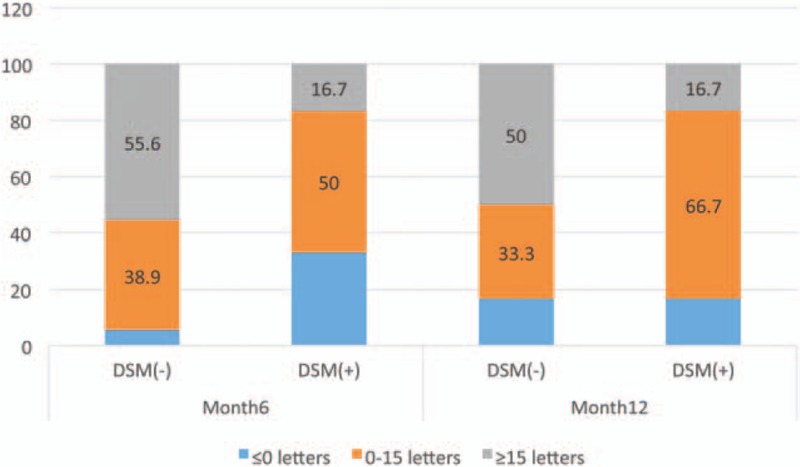
Percentage of eyes gaining ≤ 0 ETDRS, 0–15 ETDRS, and ≥15 ETDRS letters at months 6 and 12. ETDRS = Early Treatment Diabetic Retinopathy Study.

The proportion of patients in the DSM (−) group who gained at least 15 letters from baseline is greater than that in the DSM (+) group (55.6% and 16.7%, respectively, at month 6; nominal *P* = .17) (50% and 16.7%, respectively, at month 12; nominal *P* = .13). At month 6, more patients with DSM had a BCVA reduction from baseline compared with the patients without DSM (33.3% and 5.6%, respectively, at month 6; nominal *P* = .14) [Fisher exact test] (Fig. 4).

### 3.3 Anatomical outcomes

**Figure 5:**
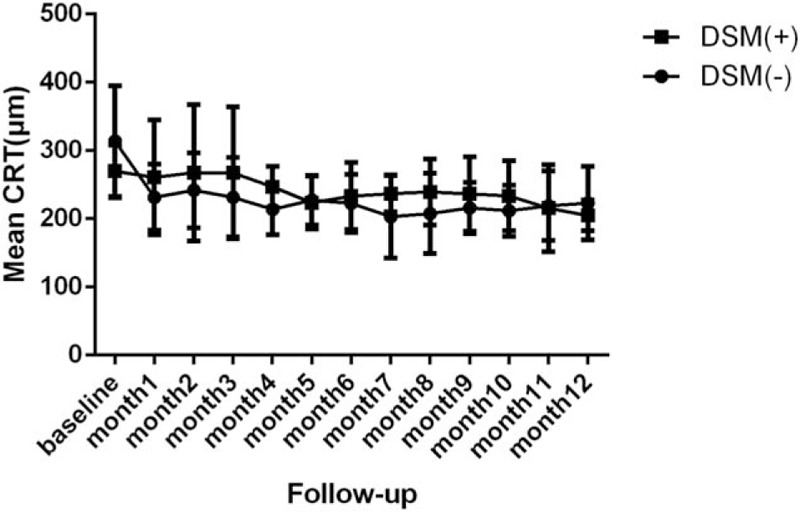
Mean central retinal thickness (CRT) in the DSM (+) and DSM (−) groups during 12 months of follow-up. DSM = dome-shaped macula.

Figure 5 shows the mean CRT of patients with and without DSM during 12 months of follow-up. At month 12, the 2 groups had a similar CRT (204.8 μm for patients with DSM and 223.1 μm for patients without DSM; *P* = .44).

**Figure 6:**
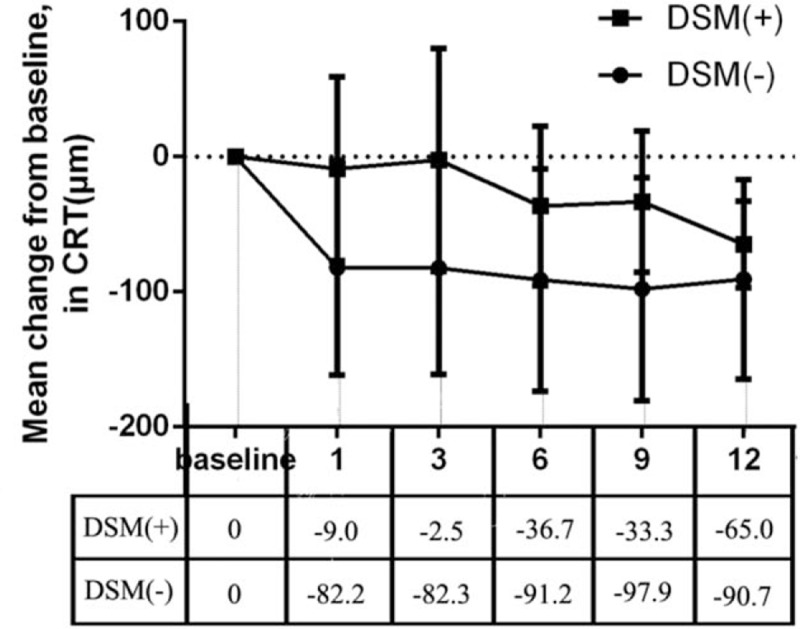
Mean change in central retinal thickness (CRT) from baseline to month 12: full analysis set.

At the first month, there was a sharp decrease in CRT for patients without DSM compared with patients with DSM (−82.2 and −9.0, respectively; *P* = .06). However, by month 12, there was no significant difference in mean CRT between the DSM (+) and DSM (−) groups (−65.0 and −90.7, respectively; *P* = .42) (Fig. 6).

### 3.4 Number of injections

The mean number of injections for the 2 groups was also evaluated in this study, and these values are shown in Table 2. The mean number of injections in the DSM (+) and DSM (−) groups was 8.83 ± 1.9 (range, 6–11) and 8.17 ± 2.6 (range, 5–12), respectively (*P* > .05).

**Table 2:**
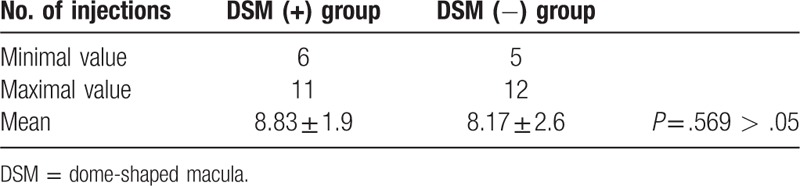
Mean number of intravitreal injections of ranibizumab in patients with and without DSM.

## 4 Discussion

Since Gaucher et al first proposed that the DSM was a special shape of pathological myopia in 2008, many related studies have been carried out. The DSM is characterized by an inward bulge of the macular region that is detected by OCT images^[8]^ (Fig. 1). Imamura explained that a DSM is the consequence of a localized thickening of the posterior sclera in the macular area seen by enhanced-depth imaging OCT.^[13]^ In our study, the DSM was observed in 6 of the 24 eyes (25%). This rate is higher than the rates of 10.7% reported by Gaucher et al^[8]^ and 9.3% reported by Ohsugi et al.^[14]^ Owing to our small sample size, the rate is not persuasive. Among the 6 eyes with DSM, 2 eyes (33.3%) had a bidirectional DSM, and 4 eyes (67.7%) had a horizontally oriented DSM. The predominance of a horizontally oriented DSM is consistent with the previous report by Ellabban et al.^[11]^

There are different views about the relationship between the rate of macular CNV and the presence of a DSM. In some studies, macular CNV has been considered a frequent DSM-related complication. Ellabban et al^[11]^ showed that CNV was present in 41.2% eyes with a DSM, while Viola et al^[12]^ reported a rate of 25%. However, Liang et al reported that the rate of macular CNV was associated with age but not with the presence of a DSM. Furthermore, they reported that the rate of retinoschisis and serous retinal detachment were markedly related to the presence of DSM.^[9]^ For pathological myopia patients, CNV is one of most common complications that typically leads to poor visual outcomes.^[15]^ As to the formation of CNV, the mechanism is not clear. Akyol et al^[16]^ considered that CNV development may be related to the choroidal and retinal blood flow changes. In contrast, Ohsugi et al^[14]^ reported that CNV formation is caused by thinning of the central sclera owing to elongation of the axial length. However, other risk factors for the formation of CNV have been reported, such as lacquer cracks, choroidal thinning,^[17]^ patchy atrophy,^[18]^ and the presence of a choroidal filling delay.^[19]^ Alternative treatments for CNV include argon laser photocoagulation,^[20]^ PDT with verteporfin (vPDT),^[21]^ macular translocation, and surgical removal.^[22]^ However, none of the therapies changed the poor long-term outcomes. At present, intravitreal anti-VEGF treatment is the first-line therapy widely used for various CNV.^[7]^

In our study, although baseline BCVA in patients with a DSM was better than that in patients without a DSM, there were no significant differences in BCVA in the 2 groups at the end of the 12-month follow-up (Fig. 2). This result is consistent with the RADIANCE study.^[23]^ Possible factors influencing the prognosis of future eyesight are the age of the patient and the size and location of CNV. A retrospective study concluded that the visual prognosis of myopic CNV was influenced by age at onset after evaluating 63 consecutive patients (73 eyes) with myopic CNV.^[24]^ Hayashi et al^[25]^ reported that younger patients with a good prognosis had smaller juxtafoveal CNV and better initial visual acuity. In Kojima's study, he revealed that patient age and CNV size determined the tendency to develop CRA, which was the main cause of a long-term decrease in visual ability in myopic CNV.^[26]^

By the end of follow-up, the mean CRT was reduced from baseline in both groups (204.8 ± 22.4 μm for patients with a DSM and 223.1 ± 53.9 μm for patients without a DSM, *P* = .42) (Fig. 4), but there was no significant difference between the mean change of the 2 groups during follow-up. The patients without a DSM showed greater reduction in the first month and then reached a steady state, whereas the patients with DSM showed a gradual reduction of CRT through the 12 months (Fig. 5). Therefore, we deduced that patients without a DSM might be more sensitive to the intravitreal ranibizumab therapy at the early stage than patients with a DSM.

The number of injections during the follow-up period was similar in both groups (8.83 injections for patients with a DSM), which is consistent with the results of the RADIANCE study.^[23]^ The similar number of injections given during follow-up suggests that the DSM feature does not alter the progress of treatment of CNV.

## 5 Conclusions

In this observational study, we evaluated visual and anatomical outcomes throughout a 12-month follow-up after intravitreal ranibizumab therapy in patients (with CNV due to PM) with and without a DSM. We found that there were no substantial differences in BCVA and CRT for patients with and without a DSM, which suggests that the DSM feature is not significantly associated with visual improvements and anatomic benefits. During intravitreal ranibizumab therapy for patients with CNV secondary to PM, the presence of a DSM does not influence therapy and treatment effects. The limitations of our study include a relatively small number of eyes in each group and the limited follow-up time. Additional studies are needed.
